# Effect of Impact Position on Repaired Composite Laminates Subjected to Multi-Impacts

**DOI:** 10.3390/ma15228039

**Published:** 2022-11-14

**Authors:** Paulo N. B. Reis, Sara R. M. Coelho, Abderrezak Bezazi

**Affiliations:** 1 Department of Mechanical Engineering, CEMMPRE, University of Coimbra, Rua Luís Reis Santos, Pinhal de Marrocos, 3030-788 Coimbra, Portugal; 2 Department of Aerospace Sciences, University of Beira Interior, 6201-001 Covilhã, Portugal; 3 Laboratoire de Mécanique Appliquée des Nouveaux Matériaux (LMANM), Université 8 Mai 1945, Guelma 24000, Algeria

**Keywords:** composite structures, adhesively bonded repair, external patch, multi-impact response, mechanical testing

## Abstract

Because the certification of aircraft structures requires significant costs and time-consuming experimental tests, all the studies carried out are strong contributions to the applicability of repairs based on adhesively bonded fibre composite patches. In this context, the main goal of this study aims to analyse the effect of the impact position on the multi-impact response of repaired composites. The results will be compared with those obtained in composites containing holes. Therefore, experimental tests will be carried out using an energy of 8 J and centrally supported samples. It was noted that the patch region proved to be very sensitive to impact due to its thickness. Full perforation occurred after two to three impacts, and to obtain higher strength it would be necessary to increase the thickness of the patch. However, depending on the location of the repair, this could bring aerodynamic problems. For the distance of 15 mm from the centre, an overlap region, the repaired laminate shows 494.7% higher impact strength than a laminate with a hole. In this case, the effect of the stress concentration is determinant in the impact fatigue life. Finally, for the 35 mm distances that are close to the border, no significant changes in impact fatigue life were observed for both the repaired laminates and those containing the hole. This leads to the conclusion that the border effect is much more significant than the presence of the hole for this distance.

## 1. Introduction

Nowadays, polymer matrix composites are increasingly replacing traditional metallic materials in most industries, especially in the aeronautic sector. This results from their excellent properties, such as fatigue strength, high specific stiffness and strength, as well as easy and fast production relative to the traditional materials [1].

However, during the operational life of an aircraft, it suffers various defects that can range from simple scratches to more complex ones such as perforations. In this context, the repair is necessary to restore the damaged component to a functional condition or, in other words, for the component to exhibit adequate strength [2,3]. Among the different types of repairs that can be used on an aircraft, bonded repairs have been gaining prominence due to the development of increasingly feasible and durable adhesives. This methodology ensures that a significant part of the load is transferred from the component to the repair through the adhesive and, consequently, the stress level in the repaired component is lower. For this purpose, literature suggests two patch configurations, both with associated advantages and disadvantages. The scarf bonding method, for example, has the advantage of minimising aerodynamic effects and bending moments, but requires suitable equipment and longer repair times [4,5,6]. Regarding the external patch method, its implementation is quite simple but leads to very high stress concentrations at the ends of the patches and very considerable aerodynamic changes that are difficult to accept [4,6,7].

Literature is abundant in numerical and experimental studies aimed at optimising patch repair [8,9,10,11,12,13]. Campilho et al. [9], for example, observed that the geometry, stacking sequence, and patch thickness are the most important parameters to optimise the mechanical performance of a repair. Focused on external patch repairs, Hu and Soutis [11] used an analytical/numerical approach to maximise their mechanical response and, for this purpose, the results were discussed at level of overlap length, patch thickness, and adhesive type. Based on experimental studies, Smahdi et al. [13] observed that scarf repairs are preferable for structures subjected to tensile loads, while overlap repairs are more suitable for compressive loads. However, according to Cheng et al. [6], a repaired structure that has been properly optimised can achieve more than 90% of its initial strength and the remaining value through hard patches.

Regarding the impact response, the studies available in the literature show that the maximum impact load increases, while the contact time and displacement are lower for repaired specimens compared to unrepaired specimens [14,15]. These results evidence that the repaired structures have higher stiffness and, consequently, higher impact strength. This evidence was confirmed by Ivañez et al. [16] from studies that used sandwich structures repaired after being subjected to ballistic impacts. On the other hand, considering the compressive strength after impact, Andrew et al. [15] only observed the advantages of repaired structures for intermediate levels of damage. Coelho et al. [17] studied the multi-impact response of laminates repaired using the external patch method and compared single- and double-patch geometries. Among the two geometries studied, the latter presented the highest impact fatigue lives, due to its higher stiffness, and confirms that geometries with mechanical properties similar to those of the parent laminate should be adopted.

However, a literature review focused on the impact response of repaired laminates, using the different available methodologies, shows that most of the studies carried out analyse the impact centred on the central position [18]. In fact, to the best of the authors’ knowledge, only two studies were found reporting the effect of impact position on repaired laminates, one referring to external patches and the other to scarf repair [18,19]. In the first study, Hou et al. [19] observed that the energy absorbed is maximum when the repaired sample is impacted at its centre and decreases when the impact points approach the external patch. In terms of damage, authors observed that the delaminated area increases with increasing distance but stabilises at 20 mm because there is an interaction between delamination and debonding within the patch. Regarding the impact position on scarf-repaired laminates, Kumari et al. [18] studied its effect using the finite element model (FEM) and by analysing the impact parameters (maximum impact load, contact time, and absorbed energy). It was found by the authors that the smallest damages occur for impacts located outside the repaired region, while the bond edge propitiates the largest.

Nevertheless, these studies have only addressed single low-velocity impact loads, but real operating conditions show that there is a high probability of repeated impacts at localised locations resulting from maintenance or in-service activities [20]. Therefore, this research aims to study the impact position effect on the multi-impact response of repaired structures, comparing the results with those obtained for the unrepaired ones. Accumulated damage seriously affects the mechanical performance of repaired composites, so it is crucial to study the effects of repeated impact. The results will be discussed based on load–time, load–displacement, and energy–time curves, as well as the different damages, in order to increase the scientific knowledge in the area of aeronautical repair and help engineers in their design. In this context, all studies are strong contributions to the applicability of adhesive repairs, because they are widely used in the aeronautical industry, but, for certification reasons, are essentially confined to non-primary structures or to primary structures whose damage is not critical enough [21,22].

## 2. Materials and Methods

Composite laminates with eight and four layers of woven bi-directional glass fibre 1110 P (110 g/m^2^) were prepared by hand lay-up with a Biresin^®^ CR122 epoxy resin and a Biresin^®^ CH122-3 hardener, both supplied by Sika (Zurich, Switzerland). In all composite laminates, the fibres were aligned in the same direction to maximise strength and stiffness, as well as to ensure the same damage typology. Literature recognises that damage and its propagation depend on the arrangement of fibres in the structure, with the stacking sequence being one of the most important factors in damage resistance [23]. This ensures that the main focus is on the repaired structure. Finally, a vacuum bag was used to eliminate any air bubbles in the laminates and, at the same time, a load of 2.5 kN was applied to guarantee a constant thickness. More details about the laminate manufacturing process can be found in [17].

**Figure 1:**
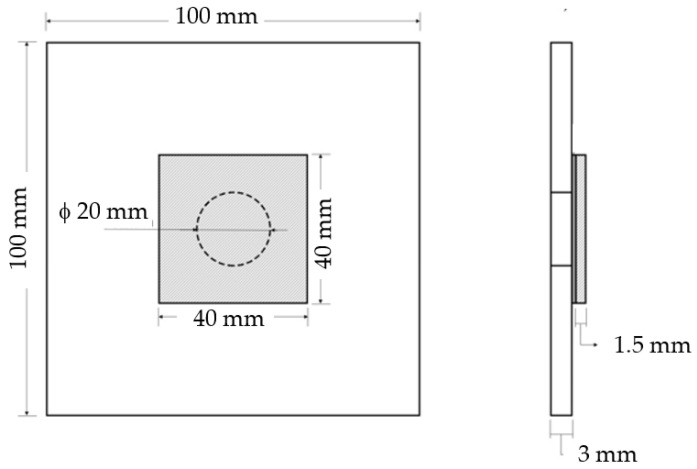
Geometry and dimensions (in mm) of the samples.

From the plates produced with dimensions of 330 × 330 × 3 [mm] and 330 × 330 × 1.5 [mm], square pieces with 100 mm and 40 mm sides were cut to produce the samples. Subsequently, in the 100 mm square pieces, holes of 20 mm in diameter were introduced to simulate the damage and, for this purpose, a special drill was used. Finally, as shown in Figure 1, the different parts were bonded with the adhesive ‘‘Araldite^®^ 420 A/B” (supplied by Antala, Barcelona, Spain). To improve the adhesion between pieces, they were previously cleaned in an ultrasonic bath and, after drying, were degreased with methyl-ethyl-ketone. The bonding procedure was concluded with the specimens being placed in an oven at 40 °C for 4 h.

**Figure 2:**
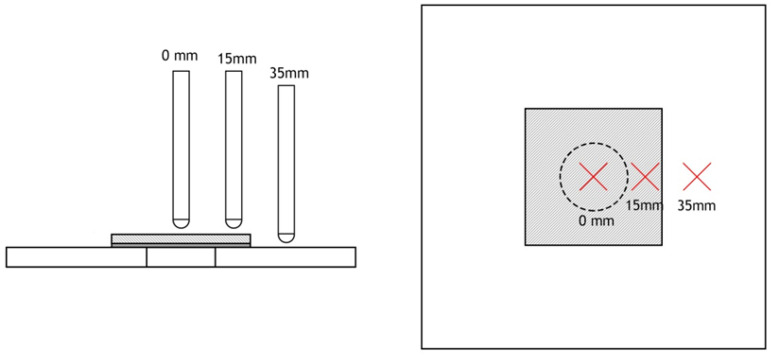
Location of the three impact points.

An IMATEK-IM10 drop weight-testing machine (conveniently described in [24]) was used to perform impact tests at room temperature and according to the procedure described in the EN ISO 6603-2 standard. The impactor used, with a diameter of 10 mm and a mass of 2.827 kg, impacts the samples that are simply supported, with dimensions of the useful section of 75 × 75 mm. An energy of 8 J was used, selected from the results published in [17], considering an adequate value to produce visible damage, but unable to penetrate the external patch due to its smaller thickness. Regarding the impact point, as shown in Figure 2, three positions were selected. The first is located at the centre of the external patch (denominated by 0 mm) and aims to assess its impact strength. Subsequently, the impact point is located 15 mm from the centre of the patch and aims to compare the impact strength between the parent and repaired laminate in the overlap region. In both cases, this point is very close to the hole and, consequently, subject to stress concentration due to its proximity. It is intended to evaluate the interaction of the repair with the stress concentration caused by the hole, and the respective impact response. Finally, the last point is located 35 mm from the centre of the repair and is close enough to the boundary conditions. In this case, the effect of boundary conditions on the impact response of repaired and damaged laminates is analysed. The damage is simulated by a 20 mm diameter hole. To ensure the uniformity of the results, each condition used at least three specimens that were subjected to several impacts with the same energy until complete perforation occurred.

## 3. Results and Discussion

**Figure 3:**
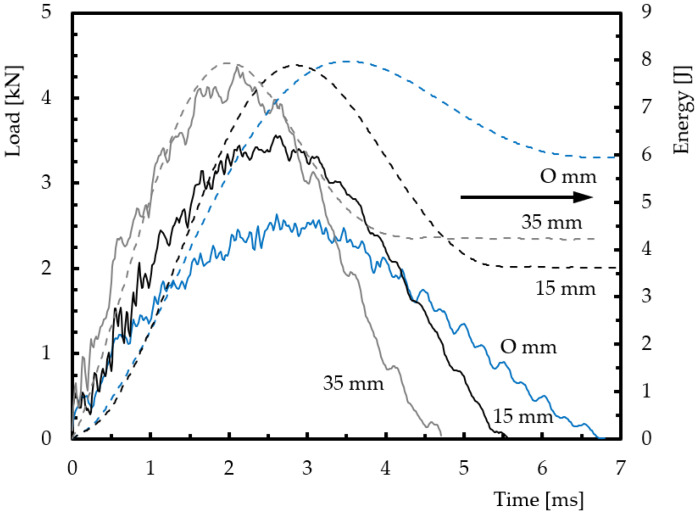
Effect of the impact point on load–time and energy–time curves obtained with an energy of 8 J and for repaired specimens.

Figure 3 shows the effect of the impact point on load–time and energy–time curves for the first impact. They represent the typical behaviour observed for all specimens and agree with those reported in the literature [15,25,26,27,28,29,30]. The oscillations observed are caused by the vibrations of the specimen [27,31].

It is possible to observe that the load increases up to a maximum value (P_max_), which depends on the impact energy and determines the failure modes triggered in the composite [25,26,27,28,29,30]. From the energy–time curves, it is noted that the energy used was not enough to perforate the laminate, because the impactor contacts the sample and is returned by it. This evidence is replicated by the profile of the energy–time curve, where the peak value represents the energy at maximum load and the beginning of the plateau at the loss of contact between impactor and sample and, consequently, represents the energy absorbed by the sample. The restored/elastic energy will be obtained by the difference between these energies [26,32], and when the collapse occurs, its value is zero, i.e., all the energy is absorbed in the form of damage.

The mean values and respective standard deviations were obtained from the previous curves and are summarised in Table 1 for all conditions analysed.

The values are compared with those obtained in specimens impacted at the centre (without a hole and 0 mm position) and those impacted at 15 and 35 mm (both with a 20 mm hole). These specimens will be designated by control and referenced by CS_0, CS_15, and CS_35, respectively. Regarding the repaired laminates, they are referenced by R_0, R_15, and R_35 and correspond to impacts at positions of 0, 15, and 35 mm from the centre of the specimen, respectively. Therefore, compared to the control samples without any damage (CS_0), the maximum impact loads are about 13% and 22.4% higher than those obtained for the unrepaired (CS_35) and repaired (R_35) specimens, respectively, and impacted at the 35 mm distance. However, the same comparison shows that the maximum load decreases by 22.2% and 6.4% when the impact point is 15 mm from the centre. In terms of maximum displacement, the comparisons reported above are the opposite for the distance of 35 mm. In this case, instead of increasing, the maximum displacement decreases by 6.1% and 14.3%, respectively. Considering the impact point of 15 mm, the displacement decreases by around 16.3% for the repaired laminate (R_35), while for the unrepaired laminates (CS_15) there is an increase of 8.2%. From this analysis, it is evident that the impact position significantly influences the maximum load and displacement values due to the change in stiffness. Amaro et al. [33] report that the maximum load and stiffness increase with the thickness, while the plate deflection is proportional to the bending stiffness, which is a function of the cube of the plate thickness. This evidence can be proved by the impact bending stiffness (IBS) values reported in Table 1. It is noticed that the repaired laminate presents higher IBS values, as well as distances closer to the edge. This property depends on the composite lay-up and is defined by the slope of the ascending branch of the load-displacement curve [34], proving to be an essential tool for evaluating the damage resistance of a composite [34,35]. Furthermore, the effect of the stress concentration caused by the hole also explains the lower values (impact load, IBS, and restored energy) obtained for the samples impacted at 15 mm from the centre. However, for distances further from the hole, the stress concentration effect diminishes, and the failure will occur when the effective stress equals the strength of the laminate [36]. Finally, this analysis is corroborated by the restored energy because, according to Amaro et al. [34], there is a relationship between it and the IBS (see Table 1).

**Figure 4:**
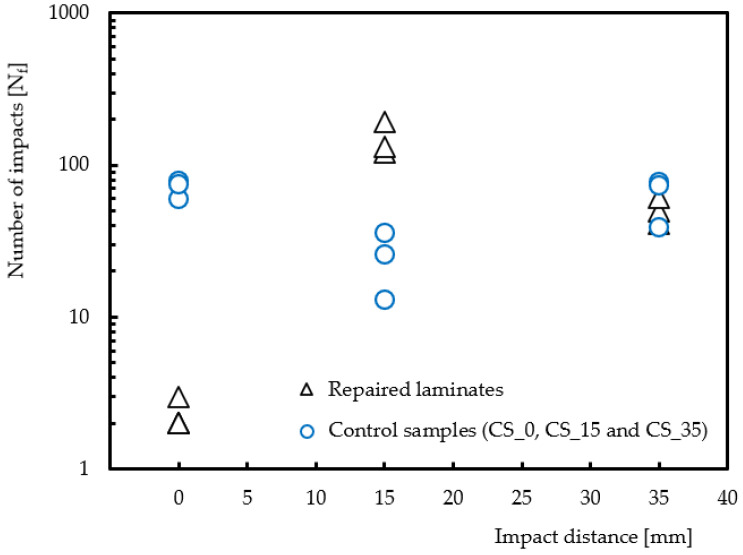
Distance effect on the number of impacts to rupture.

Figure 4 shows the number of impacts required to achieve full perforation as a function of the impact point. It is possible to observe that the patch region is the most sensitive, because, for the energy level used in this study, perforation occurs after two to three impacts. Compared to the parent laminates without holes, the impact fatigue life has an average decrease of about 96.8% due to the huge difference in stiffness [33]. In this context, higher impact strength is only achieved with thicker patches, despite the aerodynamic problems that may occur. When the impact is located 15 mm from the centre of the patch, an increase in impact strength of about 494.7% is obtained over the same position in the damaged laminates. This large increase can be explained by the higher stiffness of the repaired laminate and, simultaneously, by the stress concentration due to the hole being significantly attenuated with the patch [33,36]. Finally, when the impact occurs 35 mm from the centre of the patch, it is possible to observe similar impact fatigue lives for both laminates, highlighting that the edge effect is much more significant than the presence of the hole.

**Figure 5:**
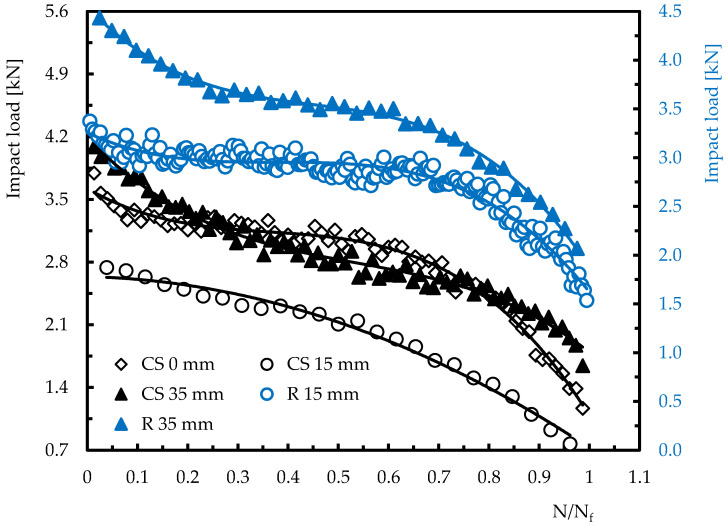
Impact load versus number of impacts for different geometries and distances.

The impact properties will be the focus of a detailed analysis to better understand the distance effect on the number of impacts to failure (N_f_). For example, Figure 5 shows how the maximum load evolves with the number of impacts for both the control laminates (in black) and those that were repaired (in blue). Only two distances were analysed for the repaired laminates, because, for the energy studied, the specimens impacted in the centre (0 mm distance) had full perforation after two to three impacts. In this figure, N/N_f_ represents the number of impacts at a given time over the total number of impacts. The impact load represents the responsiveness of a laminate and determines the type of damage introduced [25,37]. In this context, a gradual decrease in load with the number of impacts is clearly visible and, except for control specimens impacted at 15 mm from their centre (CS_15), all other configurations evolve according to a polynomial of degree three. Therefore, three stages can be well-defined, in which the first is characterised by an abrupt drop in the load, depending on the geometry and impact point, due to the severity of the first damages introduced. Afterwards, the damage slowly progresses along the second stage until it reaches saturation. Subsequently, the damage mechanisms change significantly and rapidly evolve to the final collapse, i.e., full perforation (third stage). It is also noted that control specimens without holes (CS_0) and repaired laminates impacted at 15 mm (R_15) are the ones that present a more stable second stage due to slower damage progression in these geometries.

On the other hand, the impact load profile on specimens impacted at 15 mm can be described by a second-degree polynomial, because the damage progresses much faster due to stress concentration induced by the hole. In the case of repaired laminates, this effect is mitigated by the external patch and the respective increase in stiffness promoted by it [33,36]. This analysis was also reported by Coelho et al. [17], where the decrease in the impact load was explained by the damage accumulation and consequent lower stiffness, mainly at the impact point. Finally, at the level of specimens impacted at 35 mm, despite the repaired laminates presenting slightly higher loads, both curves evolve very similarly, because the stress concentration caused by the edge is decisive in the response of both laminates.

**Figure 6:**
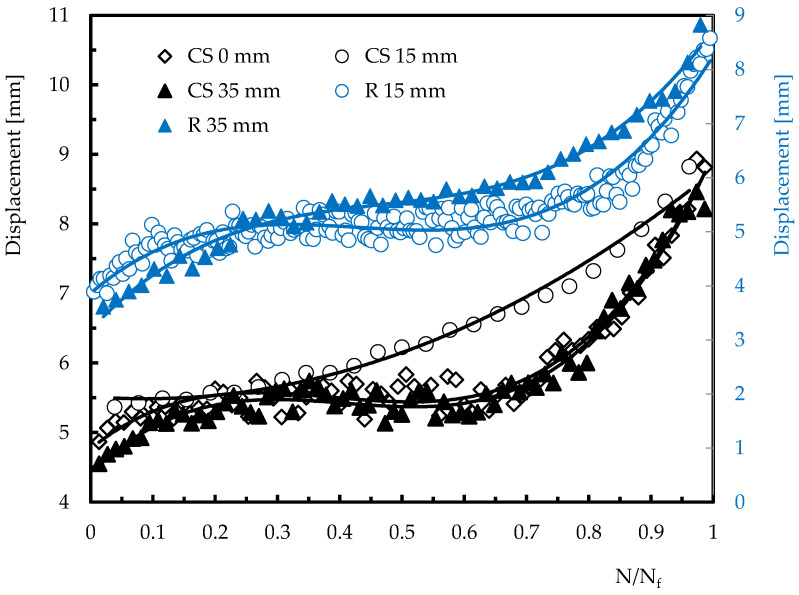
Displacement versus number of impacts for different geometries and distances.

**Figure 7:**
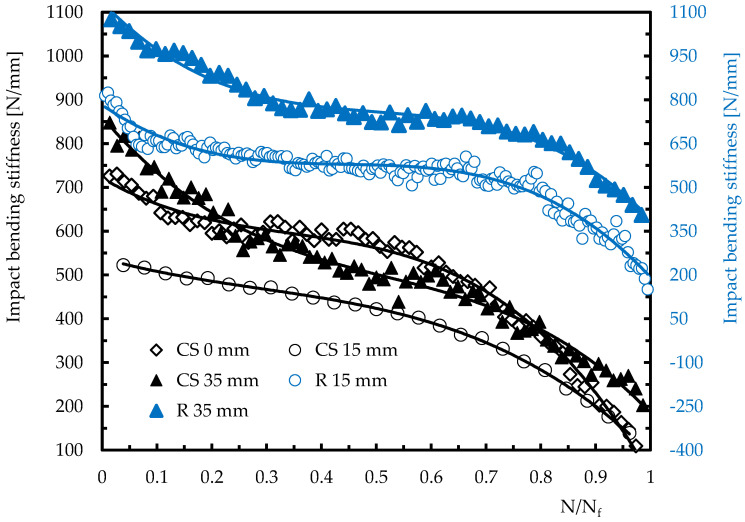
Impact bending stiffness versus number of impacts for different geometries and distances.

As shown in Figure 6, an opposite trend can be observed for displacement, which would be expected due to the accumulation of damage and consequent lower stiffness of the laminates. As observed in the previous curves (Figure 5) for the specimens impacted at 15 mm from their centre, the displacement versus number of impacts curves are also fitted by a second degree polynomial. This is a consequence of the higher severity of the damage introduced due to the proximity of the hole (stress concentration introduced by the hole). Therefore, the lower stiffness that occurred justifies the lower impact load and the higher displacement observed [38,39], and the very expressive dependence between these two parameters and the stiffness is very evident in Figure 7. The observed evolution of the impact bending stiffness (IBS) with the number of impacts presents very similar profiles to those obtained for the impact load and agrees with the literature [35,38]. In this case, as mentioned above, the stiffness loss is due to the higher damage severity introduced into the laminates. However, despite the profile observed for IBS being very similar to that obtained for the restored energy (and consequently inverse to the absorbed energy [34]), the damage is better evaluated through the IBS than through the restored energy. 

**Figure 8:**
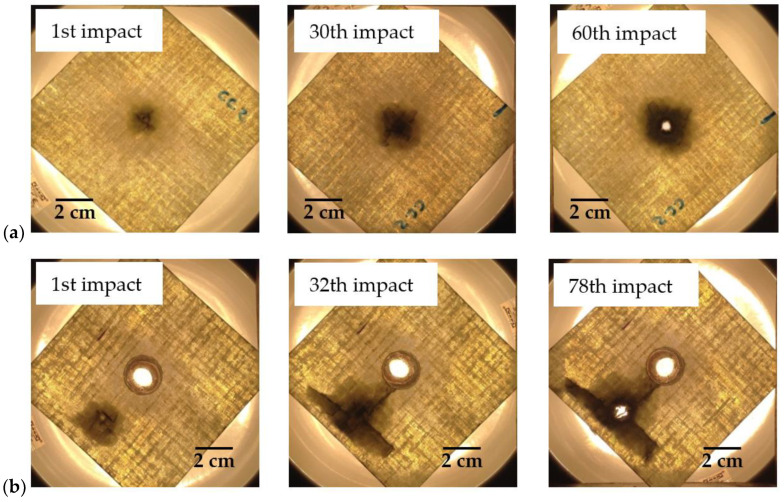
Damage evolution for control samples: (**a**) Without hole; (**b**) Impacted at 35 mm from the centre of the hole.

**Figure 9:**
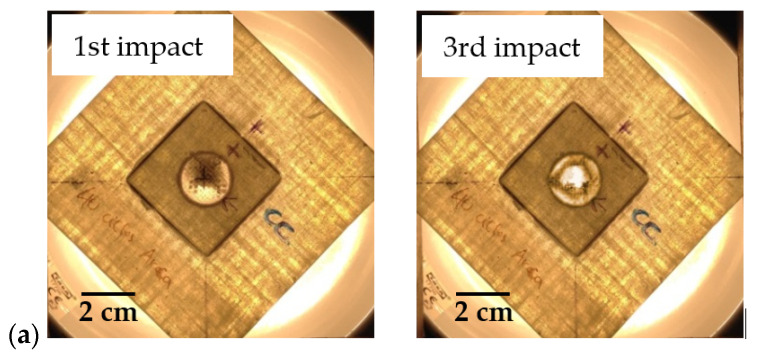

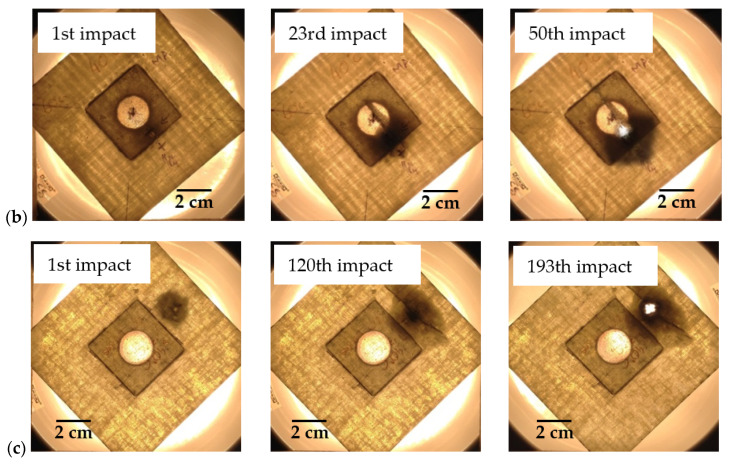
Damage evolution for repaired laminates: (**a**) Impacted at the centre; (**b**) Impacted at 15 mm from the centre; (**c**) Impacted at 35 mm from the centre.

Based on this evidence reported by Coelho et al. [17], Figure 8 and Figure 9 show some photos of the damage evolution related to the different laminates subject to multi-impacts. These figures clearly show that the damage severity is higher with increasing the number of impacts and, consequently, the stiffness of the laminates is lower [40]. Although the literature indicates that the nature of damage in impacted thick laminates is quite complex, and usually involves multiple delaminations [41], there is a consensus that the first damage mode to appear is matrix cracking [42]. For this type of sample (stiff because they are short and thick), the impact loads are high and, consequently, induce transverse shear cracks under the impactor in the upper layers [42]. Subsequently, with increasing displacement, these cracks evolve into inter- and intra-yarn cracking and, finally, into inter- and intra-ply delaminations, evidencing, through all these interactions, a very complex damage mechanism [24,43]. At a later stage, the fibres begin to fail at the impact/indentation point, but on the opposite face due to high bending stresses, and the final failure culminates in perforation when the remaining fibres can no longer withstand the impact [42]. These figures also highlight that both repaired and unrepaired laminates have very similar damage when the impact point is close to the boundary condition, which reveals that the stress concentration resulting from this situation is determinant in the whole damage process [36].

## 4. Conclusions

This study aimed to analyse the effect of impact position on the multi-impact response of repaired composite laminates. Regardless of whether the laminates have been repaired or not, impact position has been observed to have a very substantial effect on the impact strength. In terms of laminates containing a hole, for example, it has been found that the impact properties are significantly affected by its presence, but with increasing distance, the effect of stress concentration decreases, and the failure occurs when the effective stress is equal to the strength of the laminate without notches. When these laminates are repaired with external patches, the impact point also affects the impact properties and even the impact fatigue life. However, due to aerodynamic limitations imposed by the aeronautical industry, the patches are generally not very thick, and, in this case, the repaired region is very sensitive to impact loads and shorter impact fatigue lives. On the other hand, the longest impact fatigue life occurs in regions where the patch overlaps the parent laminate due to the increased stiffness of the structure promoted by the repair. Furthermore, in these overlapping regions, the patch attenuates the stress concentration effect caused by the damage/holes and the impact properties are less affected. Finally, regardless of whether the laminate is repaired or not, the stress concentration promoted by the boundary conditions determines the impact response of composite structures.

## Figures and Tables

**Table 1:** Results obtained for the different impact points.

Geometry	Load (kN)	Displacement (mm)	Restored Energy (%)	Impact Bending Stiffness (N/mm)
CS_0	3.61 (0.12)	4.9 (0.39)	64.7 (1.10)	721.3 (6.96)
CS_15	2.81 (0.06)	5.3 (0.08)	42.5 (1.41)	522.7 (15.3)
CS_35	4.08 (0.02)	4.6 (0.17)	51.1 (1.11)	844 (17.51)
R_0	2.52 (0.11)	5.1 (0.27)	28.5 (2.33)	440.2 (34.35)
R_15	3.38 (0.15)	4.1 (0.15)	49.6 (8.16)	814 (10.15)
R_35	4.42 (0.14)	4.2 (0.13)	52.1 (1.19)	1015.4 (13.57)

Average value (Standard deviation).

## Data Availability

Not applicable.
